# Internet-Based Cognitive Behavioral Therapy for Symptoms of Depression and Anxiety Among Patients With a Recent Myocardial Infarction: The U-CARE Heart Randomized Controlled Trial

**DOI:** 10.2196/jmir.9710

**Published:** 2018-03-08

**Authors:** Fredrika Norlund, Emma Wallin, Erik Martin Gustaf Olsson, John Wallert, Gunilla Burell, Louise von Essen, Claes Held

**Affiliations:** ^1^ Clinical Psychology in Healthcare Department of Women's and Children's Health Uppsala University Uppsala Sweden; ^2^ Department of Psychology Uppsala University Uppsala Sweden; ^3^ Family Medicine and Preventive Medicine Department of Public Health and Caring Sciences Uppsala University Uppsala Sweden; ^4^ Cardiology Department of Medical Sciences Uppsala University Uppsala Sweden; ^5^ Uppsala Clinical Research Center Uppsala University Uppsala Sweden

**Keywords:** eHealth, treatment adherence and compliance, patient acceptance of health care, patient selection, cardiac rehabilitation

## Abstract

**Background:**

Symptoms of depression and anxiety are common after a myocardial infarction (MI). Internet-based cognitive behavioral therapy (iCBT) has shown good results in other patient groups.

**Objective:**

The aim of this study was to evaluate the effectiveness of an iCBT treatment to reduce self-reported symptoms of depression and anxiety among patients with a recent MI.

**Methods:**

In total, 3928 patients were screened for eligibility in 25 Swedish hospitals. Of these, 239 patients (33.5%, 80/239 women, mean age 60 years) with a recent MI and symptoms of depression or anxiety were randomly allocated to a therapist-guided, 14-week iCBT treatment (n=117), or treatment as usual (TAU; n=122). The iCBT treatment was designed for post-MI patients. The primary outcome was the total score of the Hospital Anxiety and Depression Scale (HADS) 14 weeks post baseline, assessed over the internet. Treatment effect was evaluated according to the intention-to-treat principle, with multiple imputations. For the main analysis, a pooled treatment effect was estimated, controlling for age, sex, and baseline HADS.

**Results:**

There was a reduction in HADS scores over time in the total study sample (mean delta=−5.1, *P*<.001) but no difference between the study groups at follow-up (beta=−0.47, 95% CI −1.95 to 1.00, *P*=.53). Treatment adherence was low. A total of 46.2% (54/117) of the iCBT group did not complete the introductory module.

**Conclusions:**

iCBT treatment for an MI population did not result in lower levels of symptoms of depression or anxiety compared with TAU. Low treatment adherence might have influenced the result.

**Trial Registration:**

ClinicalTrials.gov NCT01504191; https://clinicaltrials.gov/ct2/show/NCT01504191 (Archived at Webcite at http://www.webcitation.org/6xWWSEQ22)

## Introduction

### Background

Symptoms of depression and anxiety are common after an acute myocardial infarction (MI). Approximately 8% to 30% of patients with a recent MI report depressive symptoms [1], and 13% to 60% of patients report anxiety symptoms [2], with anxiety often co-occurring with symptoms of depression [3]. Post-MI symptoms of depression, anxiety or both are associated with an increased risk of adverse cardiac outcomes [2,4] and reduced quality of life [5].

Several pharmacological treatment trials, with and without psychological support, have been found to reduce symptoms of depression and anxiety among patients with acute coronary syndrome [6,7]. Purely psychological treatment studies have also been effective in reducing symptoms of depression and anxiety in patients with coronary heart disease [8]. Effective treatments have been characterized by adopting techniques used in cognitive behavioral therapy (CBT) [9]. To improve access to effective support, increased engagement in eHealth solutions within the cardiac community has been called upon [10], with internet-based CBT (iCBT) representing an eHealth solution that may improve access to acceptable, effective, and cost-effective psychological treatment [11]. iCBT has been found to reduce symptoms of depression and anxiety among adults with common mental health difficulties [12]. In addition, evidence suggests that guided iCBT may improve disease-related functioning and reduce psychological distress in patients with chronic somatic conditions [13]. Furthermore, preliminary evidence suggests that iCBT may reduce symptoms of depression and anxiety for adults with high cardiovascular risk [14]. However, there is limited knowledge regarding the effectiveness and acceptability of iCBT for symptoms of depression and anxiety among MI patients recruited in a clinical setting.

### Objectives

The aim of this randomized controlled trial (RCT) was to evaluate the effectiveness of therapist-guided iCBT versus usual care in patients with a recent MI and comorbid symptoms of depression and anxiety.

## Methods

### Study Design

The U-CARE Heart study is an RCT comparing therapist-guided iCBT with treatment as usual (TAU). A study protocol, including an internal pilot study, has previously been published [15]. Patients (n=239) were recruited from 25 cardiac clinics in Sweden from September 2013 to December 2016. Outcome measurements were collected at baseline (6-10 weeks post-MI) and at post-treatment follow-up (14 weeks post baseline).

The study protocol was approved by the regional ethics committee in Uppsala (2011/217) and registered at ClinicalTrials.gov on January 5, 2012 (NCT01504191). Three protocol design modifications were made during the ongoing trial. First, the inclusion criteria threshold was lowered from ≥10 to >7 on either of the 2 Hospital Anxiety and Depression Scale (HADS) [16] subscale scores (March 5, 2014), to increase the recruitment rate (after having recruited only 7 patients). Second, minor changes were made to the introduction module after completion of the internal pilot trial including the first 20 patients [15]. Third, a mobile device version of the treatment was launched after 63 patients had been randomized to iCBT, representing 53.8% (63/117) of the total allocated to this trial arm (February 29, 2016).

### Patients

Inclusion criteria were as follows: (1) <75 years of age, (2) recent MI <3 months, and (3) score >7 on one or both of the 2 HADS subscales. Exclusion criteria were as follows: (1) scheduled for coronary artery bypass surgery, (2) unable to use computer or internet or email or mobile phone, (3) unable to read Swedish, (4) expected to live for <1 year, (5) anticipated to show poor compliance (eg, substance abuse or not showing up to the cardiac nurse visit), (6) self-reported severe depression or suicidal ideation (Montgomery-Asberg Depression Rating Scale-Self Rated [MADRS-S] total score >34 or MADRS-S item 9>3) [17], and (7) participating in another behavioral intervention trial. Patients in both study arms had access to TAU.

### Procedure

Patients were identified and screened for eligibility during a routine visit to a cardiac nurse at 1-8 weeks following their MI. Nurses provided brief trial information and logged all consecutive patients matching the inclusion criteria. U-CARE research staff at the coordinating center (Uppsala) called eligible patients to provide further study information. Written information and an informed consent form were sent to patients via postal service. Patients providing informed consent subsequently received an email with a username and password to access a secure internet-based portal to complete the Web-based baseline assessments. Patients reporting symptoms of depression or anxiety >7 on 1 or both of the 2 HADS subscales were randomized to iCBT or TAU. Patients were randomly assigned (stratified by the clinical recruiting center) with a 1:1 allocation, using a computer-generated code. Randomization occurred automatically in the internet-based portal, with patients receiving an email to inform them of condition assignment.

Patients indicating severe depression or suicidal ideation were contacted via phone and referred to appropriate care and excluded from the trial. Patients who did not complete the Web-based baseline or follow-up assessment were reminded by SMS text messages (short message service, SMS), with research staff blind to group allocation telephoning patients who did not complete the assessment within 1 week of receiving the SMS reminder. Paper-and-pencil assessment forms were sent to patients on request or if they were not reached by telephone.

**Table 1:** Description of the internet-based cognitive behavioral treatment.

Modules	Psychoeducation	Examples of homework assignments
Introduction	The CBT^a^ model	Define personal problems and goals
	Common emotional reactions post-MI^b^	
Managing worry	Worry awareness	Exposure for worry with response prevention
	Rational for worry exposure	
Fear and avoidance	Basic principles for fear and exposure	Graded exposure in situations related to cardiac or other fears
	Rational for graded exposures	
Behavioral activation	Vicious circles in depression	Self-monitoring of mood and daily activities
	Rational for behavioral activation	Plan daily activities
Problem solving	Basic problem-solving skills	Apply problem-solving skills
Communication skills	Basic communication skills and relationship-strengthening skills	Apply communication and relationship-strengthening skills
Applied relaxation training	Applied relaxation training protocol	Practice according to relaxation training protocol
Managing negative thoughts	Cognitive restructuring	Self-monitor thoughts and apply cognitive restructuring skills
Coping with insomnia	Sleep hygiene, stimulus control, and sleep restriction	Self-monitor sleep and apply sleep restriction
Values in life	Personal values and quality of life	Formulate personal values and create an action plan according to them
Relapse prevention	Relapse prevention of depression and anxiety	Identify personal preventive strategies

^a^CBT: cognitive behavioral therapy.

^b^MI: myocardial infarction.

### Interventions

#### Internet-Based Cognitive Behavioral Therapy

The treatment consisted of a 14-week, therapist-guided, tailored CBT intervention delivered via a secure internet-based portal (U-CARE-portal). See Multimedia Appendix 1 for a sitemap and Multimedia Appendix 2 for a screenshot of the portal. The treatment was developed by licensed psychologists, in consultation with patients with a history of depression and anxiety post-MI. The treatment included 10 modules with different themes, adapted to MI patients (Table 1). The introduction module was compulsory, and thereafter, patients were able to choose which modules to work with, as informed by previous research suggesting tailored iCBT provides patients with more control while maintaining treatment quality [18]. Each module contained 2 to 4 treatment steps, with each step including a PDF with text-based psychoeducation, and 1 to 2 homework assignments. Patients were recommended to work with 1 step per week during the treatment period. Homework assignments consisted of self-monitoring, skills training, and engagement in exercises based on CBT techniques (Table 1). Modules were considered complete when all homework assignments within a module were sent to the therapist for feedback. In addition, the iCBT treatment included a library with supplementary material and video clips of interviews conducted with post-MI patients concerning coping with common psychological reactions post-MI. Patients also had access to a discussion board where they could communicate with other patients randomized to the treatment arm.

#### Therapist Support in Internet-Based Cognitive Behavioral Therapy

Each patient was assigned 1 of the 3 available therapists, who were all licensed psychologists specialized in CBT. Each therapist provided feedback on homework assignments via the portal. The purpose of feedback was to express empathy, encourage work with the treatment, and reinforce treatment activity, all of which has been found to correlate with adherence and outcome [19]. Patients were able to contact their therapist at any time, with therapist responses provided within 48 hours. Patients who were inactive for more than 1 week were contacted by their therapist via telephone, with SMS reminders sent if they were unable to be reached via telephone. Motivational interviewing techniques were used during telephone calls to resolve any identified barriers regarding treatment inactivity. Occasionally, telephone calls included explanations regarding treatment module content; however, calls were not therapeutic and focused on working directly with the material. Telephone call duration ranged between 5 and 30 min. Furthermore, technical support provided by research staff (blinded to allocation) was available via telephone and email.

#### Treatment as Usual

Patients were treated by the local health care system according to international guidelines regardless of treatment allocation. TAU usually includes secondary preventive interventions (eg, information about risk factors and lifestyle changes), cardiac rehabilitation activities (eg, physical exercise), and psychosocial support (eg, counseling if available). Psychotropic medication was not restricted by study participation.

### Assessments

#### Patient Characteristics

Sociodemographic data were obtained from baseline assessments. Medical and risk factor data were obtained from the SWEDEHEART (Swedish Web-system for Enhancement and Development of Evidence-based care in Heart disease Evaluated According to Recommended Therapies, a Swedish nation-wide quality register) databases RIKS-HIA (Register of Information and Knowledge about Swedish Heart Intensive Care Admissions) and SEPHIA (Secondary Prevention after Heart Intensive Care Admission), covering over 90% of all MIs in Sweden [20].

#### Primary Outcome

HADS-total score (HADS-T) was the primary outcome measure of self-reported symptoms of depression and anxiety, consisting of 14 items divided equally on 2 subscales: anxiety (HADS-A) and depression (HADS-D). Each item is rated on a 4-point Likert scale, resulting in a total score of 42. Higher scores indicate more severe symptoms, with scores above 7 on either subscale indicating mild symptoms [16]. HADS is a reliable and valid measurement of symptom severity and can detect cases of depression and anxiety in different populations [21]. Several studies support the validity of Web-based administration of HADS [22].

#### Secondary Outcomes

MADRS-S was used to screen for severe depression and suicidal ideation before inclusion and as a secondary outcome measure of self-reported depression [17]. The scale consists of 9 items, with each item rated on a 7-point Likert scale, with a total score of 54. Higher scores indicate a higher level of depressive symptoms. The MADRS-S has adequate psychometric properties administered via both paper-and-pencil assessment and the internet [23].

The Behavioral Activation for Depression Scale-Short Form (BADS-SF) was used as a secondary outcome measure of self-reported symptoms of depression [24]. The scale has 9 items and 2 subscales: avoidance and activation. Each item is rated on a 7-point Likert scale, with a total score of 54. Higher scores indicate less symptoms of depression. The BADS-SF has shown good reliability and validity, predictive validity, and ability to detect clinically relevant changes [24].

Cardiac anxiety was assessed by the Cardiac Anxiety Questionnaire (CAQ) [25]. The scale consists of 18 items and 3 subscales: fear, avoidance, and focus on cardiac-related stimuli and sensations. Each item is rated on a 4-point Likert scale resulting in a total score of 72, with higher scores indicating a higher level of heart-focused anxiety. CAQ has shown reliability and validity among cardiac patients [25].

Adherence was defined as the proportion of treated patients completing the prescribed amount of content within the treatment period [26]. More data on usage and user experience of the intervention were collected, and a detailed analysis of these is presented elsewhere [27].

### Statistical Analysis

A statistical analysis plan prepared in line with the CONSORT (Consolidated Standards of Reporting Trials) 2010 statement was completed before the trial database was locked, and treatment allocation was disclosed. No interim analysis was performed. The study had enough patients (n>126) to detect a medium effect size (Cohen's *d*=0.5) with the power of 80 at alpha level .05.

Descriptive statistics are presented as mean (SD) or count (%) by treatment group, unless otherwise specified.

The main analysis was conducted according to the intention-to-treat (ITT) principle for all outcomes. Multiple linear modeling was used to analyze the treatment effect on outcomes. Treatment allocation was entered as an independent variable, and HADS-T at follow-up was entered as a dependent variable. To achieve increased precision, age, gender, and baseline HADS-T were entered as covariates. In case of a nonsignificant treatment effect from the main analysis, 2 exploratory analyses with the HADS subscales (HADS-A and HADS-D) as separate outcomes were conducted. For the HADS-A analysis, only patients scoring >7 on the HADS-A subscale at baseline were included, with the corresponding selection applied to the HADS-D analysis. Thus, it was possible for patients to be included in both analyses if they score >7 on both subscales at baseline.

ITT analyses were preceded by multiple imputation via chained equations and predictive mean matching [28]. This was done because (1) there were 11.7% (28/239) with missing values in the main outcome, (2) we could not expect values missing completely at random, and (3) preplanned analyses included multiple outcomes. The imputation model included main effects and the following prespecified interactions: age*treatment and sex*treatment. Moreover, 100 imputed datasets were created. The linear model was thereafter fit to each of these datasets, and resulting effect estimates were pooled using Rubin rules [29]. Sensitivity analyses of HADS-T were conducted on observed data. Supplementary analyses of HADS-T were performed based on per protocol (PP) data from all patients who had completed at least one homework assignment. Secondary outcomes were analyzed using ITT only. We report effect estimates as pooled adjusted point estimates (beta) with 95% CI. Paired *t* tests were performed for all outcomes (baseline vs follow-up) to assess change over time. The relationship between number of completed homework assignments and changes in HADS-T over time was calculated with Spearman rank-order correlation. Statistical significance was set to 5% (2-tailed).

Analyses were performed in R version 3.4.0 (R Foundation for Statistical Computing, Vienna, Austria) [30] using packages base, foreign, ggplot2, mice, miceadds, MKmisc, stats, tableone, and VIM, and IBM SPSS version 22 (IBM Corp, Armonk, NY).

## Results

### Recruitment

**Figure 1:**
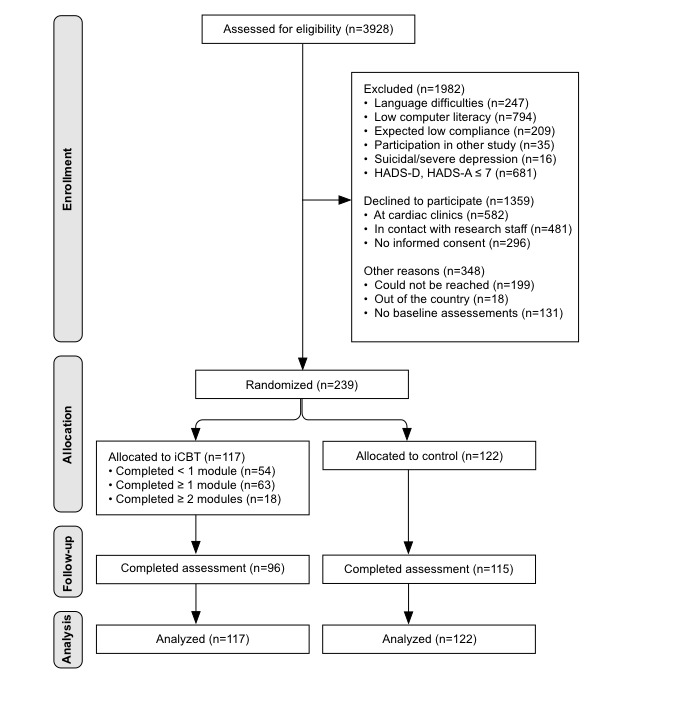
Flowchart of patients through the U-CARE Heart trial. iCBT: Internet-based cognitive behavioral therapy; HADS-D: Hospital Anxiety and Depression Scale-Depression subscale; HADS-A: Hospital Anxiety and Depression Scale-Anxiety subscale.

During the 40-month recruitment period, 3928 patients were screened for eligibility, with a total of 239 (6.08% (239/3928) of all screened) randomized. Of these, 10.9% (26/239) were included based on HADS-D only, 38.1% (91/239) based on HADS-A only, and 51.0% (122/239) based on both subscales. The main reasons for exclusion were the following: being unable/unwilling to use the internet or mobile phone, followed by scoring <8 on both HADS subscales and language difficulties. In total, 34.6% (1359/3928) declined participation or did not return the informed consent form. Follow-up assessment was completed by 88.3% (211/239) of all patients, with a significantly higher percentage of completers in the control group (94.3%, 115/122) compared with the treatment group (82.1%, 96/117; Pearson χ^2^_1_=8.6, *P*=.003). See Figure 1 for a study flowchart.

### Patient Characteristics

Baseline patient characteristics were similar in both groups (Table 2). On average, patients were 59.6 years of age (SD 8.49), 33.5% (80/239) were women, 41.8% (100/239) had university level of education, 60.3% (144/239) were employed, and 18.0% (43/239) were taking antidepressant and/or anxiolytic medication in both groups. The corresponding percentages after treatment was 19.6% (18/92; 25 missing values) in the treatment group and 15.6% (18/115; 7 missing values) in the control group (*P*=.37).

**Table 2:** Patient characteristics. Observed data (no imputations).

Characteristics			iCBT^a^ (n=117)	TAU^b^ (n=122)	Missing n (%)
**Sociodemographic**					
	Age in years, mean (SD)		58.4 (9.0)	60.8 (7.8)	
	Women, n (%)		44 (37.6)	36 (29.5)	
	**Occupation, n (%)**				14 (5.8)
		Employed	78 (66.7)	66 (54.1)	
		Unemployed	4 (3.4)	2 (1.6)	
		Retired	33 (28.2)	37 (30.3)	
		Sick leave	2 (1.7)	1 (0.8)	
		Other	0 (0.0)	2 (1.6)	
	**Highest level of education, n (%)**				
		Elementary	22 (18.8)	26 (21.3)	
		High school	45 (38.5)	46 (37.7)	
		University	50 (42.7)	50 (41.0)	
	In a relationship, n (%)		99 (84.6)	101 (82.8)	
	Children in the household, n (%)		43 (36.8)	34 (27.9)	
	Country of birth other than Sweden, n (%)		21 (17.9)	15 (12.3)	
	Smoking, n (%)		6 (5.1)	8 (6.6)	
	Alcohol, standard drinks/week (SD)		5.7 (13.7)	5.5 (6.1)	
	**Leisure time physical activity, n (%)**				
		High activity	19 (16.2)	24 (19.7)	
		Moderate activity	52 (44.4)	65 (53.3)	
		Low activity	37 (31.6)	26 (21.3)	
		Sedentary lifestyle	9 (7.7)	7 (5.7)	
	**Psychotropic medicine, n (%)**				
		Anxiolytics	10 (8.5)	7 (5.7)	
		Antidepressants	11 (9.4)	15 (12.3)	
		No	98 (83.8)	102 (83.6)	
	Other current counseling, n (%)		30 (25.7)	28 (22.9)	
**Medical history**					
	Myocardial infarction, n (%)		19 (16.2)	13 (10.7)	10 (4.2)
	Diabetes, n (%)		21 (17.9)	19 (15.6)	9 (3.7)
	Hypertension, n (%)		42 (35.9)	51 (41.8)	9 (3.7)
	Hyperlipidemia, n (%)		26 (22.2)	27 (22.1)	9 (3.7)
	Stroke, n (%)		0 (0.0)	4 (3.3)	4 (1.7)
	Heart failure, n (%)		4 (3.4)	2 (1.6)	16 (6.7)
**Cardiac status and medication**					
	Any angina/chest pain, n (%)		34 (29.0)	32 (26.2)	30 (12.6)
	Blood pressure <140/90, n (%)		66 (56.4)	78 (63.9)	31 (13.0)
	Body mass index, mean (SD), kg/m^2^		27.8 (5.0)	27.4 (4.0)	18 (7.5)
	Beta-blockers at discharge, n (%)		104 (88.9)	106 (86.9)	9 (3.8)
	Statins at discharge, n (%)		110 (94.0)	115 (94.3)	9 (3.8)
	ACE^c^ inhibitor/ARB^d^ at discharge, n (%)		89 (76.1)	96 (78.7)	9 (3.8)
	DAPT^e^ at discharge, n (%)		107 (91.4)	107 (87.7)	10 (4.2)

^a^iCBT: internet-based cognitive behavioral therapy.

^b^TAU: treatment as usual.

^c^ACE:angiotensin-converting enzyme.

^d^ARB: angiotensin receptor blocker.

^e^DAPT: dual antiplatelet therapy.

In total, 33.1% (79/239) were sedentary or reported low levels of exercise, 16.7% (40/239) had previous diabetes mellitus, and 13.4% (32/239) had a previous MI. At baseline, 25.7% (30/117) in the iCBT group and 22.9% (28/122) in the control group had regular contact with a counselor within TAU. The corresponding percentage at follow-up was 21.1% (19/90; 27 missing values) in the iCBT group and 27.2% (31/114; 8 missing values) in the control group (*P*=.33).

### Primary Outcomes

There was no difference in HADS-T scores at baseline between the iCBT and the control group (*t*_237_=0.56, *P*=.85). There was a general reduction in HADS-T over time in the total study sample (mean delta=−5.1; *t*_237_=12.92, *P*<.001).

The main analysis showed no effect of treatment on HADS-T at follow-up (beta=−0.47, 95% CI −1.95 to 1.00, *P*=.53). Furthermore, the main analysis showed that men scored lower on HADS-T compared with women at follow-up (beta=−2.04, 95% CI −3.60 to −0.47], *P*=.01), and there was a borderline significant reduction in HADS-T per unit increase in age (beta=−0.08, 95% CI −0.16 to 0.01, *P*=.09) at follow-up. There was no interaction between treatment and sex, or treatment and age, on HADS-T (*P* for both >0.19). Congruent with the main analysis, separate exploratory analyses showed no effect of treatment on either HADS-A or HADS-D subscales (Table 3).

Results of the sensitivity analyses were consistent with the ITT analysis. Both the PP analysis (beta=−0.87, 95% CI −2.47 to 0.72, *P*=.28) and the analysis with observed data (beta=−0.55, 95% CI −2.04 to 0.93], *P*=.46) with HADS-T as the outcome yielded no effect of treatment (Table 3).

### Secondary Outcomes

Additional multiple linear models showed no effect of treatment on the secondary outcomes MADRS-S, CAQ, or BADS-SF at follow-up (Table 3).

### Adverse Events

Two patients in the iCBT group and 3 patients in the control group reported severe depression (MADRS-S>34) or suicidal ideation (MADRS-S item 9 >3) at follow-up.

### Adherence

**Figure 2:**
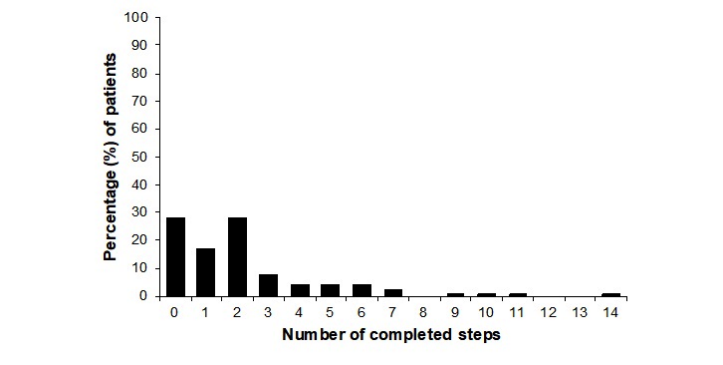
Proportion of patients completing different number of steps in the internet-based cognitive behavioral therapy.

Treatment adherence was low, with 46.2% (54/117) of the iCBT group not completing the introductory module, 38.4% (45/117) completing the introductory module only, and 15.4% (18/117) completing additional modules (Figure 2). Furthermore, only 0.9% (1/117) adhered to the treatment [26] by completing the recommended number of 14 steps within the 14-week treatment period. The number of completed homework assignments was not associated with change in HADS-T at follow-up, *r*_s_=.07, *P*=.53.

**Table 3:** Outcomes at baseline and follow-up, change scores, and treatment effects. Mean (SD) and change are calculated from observed data. Effect estimates (beta) are pooled adjusted coefficients for treatment (internet-based cognitive behavioral therapy, iCBT) versus control (treatment as usual) on follow-up outcomes adjusted for sex, age, and baseline levels of the respective outcomes after multiple imputation.

Outcome		Baseline, mean (SD)	Follow-up, mean (SD)	Change	Effect, Beta (95% CI)	*P* value
**HADS-T^a^**					−.47 (−1.95 to 1.00)	.53
	iCBT	18.3 (4.9)	12.8 (5.9)	−5.5		
	Control	18.6 (5.0)	13.6 (6.8)	−5.0		
**HADS-A^b^**					−.09 (−0.91 to 0.72)	.82
	iCBT	10.9 (2.4)	7.4 (3.2)	−3.5		
	Control	10.8 (2.5)	7.3 (3.7)	−3.5		
**HADS-D^c^**					−.45 (−1.34 to 0.44)	.32
	iCBT	9.9 (2.2)	6.6 (3.3)	−3.3		
	Control	10.3 (2.5)	8.0 (3.8)	−2.3		
**MADRS-S^d^**					−.58 (−2.20 to 1.04)	.48
	iCBT	14.8 (6.4)	12.0 (7.2)	−2.8		
	Control	15.9 (7.2)	13.3 (7.6)	−2.6		
**CAQ^e^**					−.73 (−2.83 to 1.38)	.50
	iCBT	26.1 (10.3)	21.5 (10.2)	−5.4		
	Control	25.3 (10.8)	22.0 (11.4)	−3.3		
**BADS-SF^f^**					−.50 (−2.31 to 1.30)	.58
	iCBT	21.2 (6.1)	21.4 (6.9)	0.2		
	Control	21.4 (7.7)	21.6 (7.2)	0.2		

^a^HADS-T: Hospital Anxiety and Depression Scale total score.

^b^HADS-A: Hospital Anxiety and Depression Scale anxiety subscale.

^c^HADS-D: Hospital Anxiety and Depression Scale depression subscale.

^d^MADRS-S: The Montgomery-Asberg Depression Rating Scale-Self Rated.

^e^CAQ: Cardiac Anxiety Questionnaire.

^f^BADS-SF: Behavioral Activation for Depression Scale-Short Form.

## Discussion

### Principal Findings

In this RCT, we evaluated the effectiveness of a therapist-guided, tailored iCBT treatment compared with TAU to reduce symptoms of depression and anxiety among recent MI patients. Both groups reported a decreased level of symptoms of depression and anxiety over time to a similar extent, with no difference between groups at follow-up. Adherence was low compared with other tailored iCBT interventions for depression and anxiety [26], indicating most patients allocated to iCBT received only a small treatment dose.

Overall, 6.08% (239/3928) of the screened patients were randomized. The main reasons for exclusion were reported as being unable or unwilling to use internet or mobile phone, HADS score below the inclusion threshold (<8), and language difficulties. Furthermore, a substantial number of patients screened for eligibility declined to participate. Reasons for declining are not fully known, but might include low perceived need for help or a preference for other treatment alternatives. Low interest in iCBT treatment among cardiac patients has also been reported previously. The InterHerz study [31], which resembles the U-CARE Heart RCT, ended prematurely because of low recruitment rates (12 patients in 6 months; personal communication October 1, 2017 with Professor Nadine Messerli-Bürgy). Negative attitudes toward, and low intentions to use, internet-based psychological interventions have been reported previously in other populations [32,33]. Access to face-to-face counseling and psychotropic medicine is readily available and is of good quality in standard MI care in Sweden, with an estimated 95% of cardiac clinics in Sweden assessing and referring patients with mental health difficulties to appropriate care [34]. As such, a low interest in iCBT interventions among cardiac patients may be expected, which in turn may be a barrier, or at least a challenge, for implementation in routine care.

Previous findings suggest iCBT as an effective treatment for comorbid symptoms of depression and anxiety in patients with somatic conditions [13], and effective psychological interventions for emotional distress related to coronary heart disease are characterized by CBT techniques [9]. Given this, the lack of effect of the iCBT intervention found in this study may have several explanations. Two factors might be low treatment adherence in the intervention group and a significant spontaneous improvement in the control group. Most iCBT studies with positive results are efficacy studies based on self-referral by people seeking help on the internet. In this study, we recruited patients within a routine care setting using screening methods. However, treatment adherence to iCBT interventions has been found to be lower in effectiveness studies in primary care samples compared with samples recruited from Web-based self-referral [35,36]. It is likely that patients actively seeking out and self-referring to iCBT are more prone to stay active in treatment compared with those who are screened and offered participation. Indeed, some of the patients in this study reported that their strongest reason for joining the study was to assist in research rather than seeking help for their depression or anxiety. In addition, reporting severe depressive symptoms was an exclusion criterion.

Another important factor that may have influenced treatment adherence was related to iCBT characteristics. The treatment and U-CARE portal used to deliver it were developed in consultation with patients with personal experience of depression and anxiety post-MI. In spite of this effort, the content and design of the intervention might not have been adjusted enough to end users’ needs, for example, in terms of relevance and workload. Indeed, treatment burden and failure to tailor content adequately are associated with negative iCBT user experience [37]. Moreover, therapist support has been shown to significantly improve iCBT treatment adherence and effect [38]. However, the amount of support needed in different populations may vary, with some patient populations potentially benefitting from more extensive support. Indeed, extending iCBT support through additional weekly telephone calls has been found to improve treatment adherence [39]. As such, real-time therapist support via telephone might have helped patients engage with and adhere to treatment over time. Furthermore, some previous successful psychological interventions (with high adherence) for cardiac patients [40-42] have been group-based CBT with a process-oriented focus. It remains to be investigated in a randomized trial if process-oriented, group-based formats are necessary in psychological interventions for cardiac patients.

Patient characteristics may affect treatment adherence. The mean age of patients in this study was >10 years lower than the average MI population, but higher compared with other iCBT studies of patients with depression and anxiety [35,43,44]. Older age is correlated with lower computer literacy [45]. It is possible that patients experiencing technological difficulties were less active in treatment. Furthermore, the level of education was somewhat lower compared with other iCBT studies (40% university level vs 50-60%) [35,43,44], a factor further associated with low adherence to psychological treatment [46].

Both groups reported improved psychological symptoms over time, with regression to the mean potentially explaining this pattern. In addition, a substantial spontaneous improvement has been reported for MI patients in symptoms of both anxiety and depression over time [47]. Our patients were recruited about 10 weeks post-MI to avoid spontaneous recovery diluting any treatment effects. However, this recruitment strategy may have resulted in patients finding other ways to improve their psychological well-being. Moreover, more patients in the control group than in the iCBT group reported initiating a contact with a local counselor during the study period, but the difference was not significant.

### Strengths and Limitations

This trial recruited patients from 25 hospitals in both rural and urban areas in Sweden. The content and design of the portal and the treatment were developed in consultation with patients with personal experience of emotional distress post-MI to increase acceptability, relevance, and usability. We prepared a detailed statistical analysis plan and prespecified adjustment by covariates to ensure a transparent analysis procedure [48]. We have provided detailed descriptions of the intervention and its delivery, in line with recent reporting guidelines [49], enabling comparison with other iCBT treatments targeting cardiac patients. Therapist support was provided by licensed psychologists, specialized in the CBT methodology. Despite all efforts to develop a user-friendly and relevant iCBT treatment, adherence to treatment was low. Given the obtained dose of treatment was low, the effect of the treatment might be difficult to evaluate. The PP analysis did not differ from the ITT analysis. However, our definition of PP may be criticized of being too liberal (completion of only 1 homework assignment). Moreover, HADS was developed as a screening measure and might not be sensitive enough to detect minor changes over time. However, none of the more sensitive secondary outcomes assessments indicated an effect. Diagnostic interviews might have been a more valid assessment of symptoms of depression and anxiety. Furthermore, the initial cut-off of >10 in any of the HADS subscales was lowered early in the study to >7 to increase recruitment rate. Patients reporting a low level of depression and anxiety have less room for improvement, resulting in a reduced likelihood of detecting a treatment effect [50]. This might also have resulted in inclusion of patients experiencing a low level of emotional distress, and consequently low perceived need for psychological help.

### Future Directions

Effective and accessible psychological treatments are important, given symptoms of depression and anxiety are common post-MI. Despite the success of iCBT trials [13], using self-referral recruitment methods for patients with a range of comorbid physical and mental conditions, alongside calls for the widespread implementation of eHealth interventions for cardiac populations [10], this study questions the promise of iCBT for MI patients recruited at cardiac clinics. As such, future research should examine the potential differences in terms of iCBT acceptability between populations recruited via self-referral versus clinical settings. Furthermore, increased efforts are needed to better understand how to improve treatment adherence. Such efforts may include exploratory studies investigating factors related to treatment acceptability. In interviews with participants in this study, some challenges have been identified. These are described elsewhere [27]. Finally, our results support the notion that systematic development and feasibility testing, in close collaboration with potential end users, should be undertaken to improve treatment relevance and acceptability [51]. Although this study was preceded by both semistructured feasibility testing and an internal pilot study, this was apparently not sufficient.

### Conclusions

In a randomized trial, we evaluated the effects of a therapist-guided, tailored iCBT intervention for depression and anxiety versus TAU among recent MI. Both groups reported less emotional distress after treatment, but iCBT did not significantly reduce symptoms of depression or anxiety in comparison with TAU. This lack of difference in treatment outcome may be explained by low treatment adherence, with further investigation into reasons for poor treatment adherence warranted. This study suggests that further research is required into the acceptability and feasibility of iCBT for an MI population before wide-scale implementation of similar eHealth solutions for this patient group.

## Appendix

Multimedia Appendix 1 Sitemap of the Web-based portal.

Multimedia Appendix 2 Screenshot of the U-CARE Heart portal.

Multimedia Appendix 3 CONSORT E-HEALTH checklist (v 1.6.1).

## Abbreviations

BADS-SF: Behavioral Activation for Depression Scale-Short Form
CAQ: Cardiac Anxiety Questionnaire
CBT: cognitive behavioral therapy
HADS: Hospital Anxiety and Depression Scale
HADS-A: HADS-anxiety score
HADS-D: HADS-depression score
HADS-T: HADS-total score
iCBT: internet-based cognitive behavioral therapy
ITT: intention-to-treat
MADRS-S: Montgomery-Asberg Depression Rating Scale-Self Rated
MI: myocardial infarction
PP: per protocol
RCT: randomized controlled trial
SMS: short message service
TAU: treatment as usual

## References

[1] Thombs BD, Bass EB, Ford DE, Stewart KJ, Tsilidis KK, Patel U, Fauerbach JA, Bush DE, Ziegelstein RC (2006). Prevalence of depression in survivors of acute myocardial infarction. J Gen Intern Med.

[2] Roest AM, Martens EJ, Denollet J, de Jonge P (2010). Prognostic association of anxiety post myocardial infarction with mortality and new cardiac events: a meta-analysis. Psychosom Med.

[3] Lane D, Carroll D, Ring C, Beevers DG, Lip GY (2002). The prevalence and persistence of depression and anxiety following myocardial infarction. Br J Health Psychol.

[4] Meijer A, Conradi HJ, Bos EH, Thombs BD, van Melle JP, de Jonge P (2011). Prognostic association of depression following myocardial infarction with mortality and cardiovascular events: a meta-analysis of 25 years of research. Gen Hosp Psychiatry.

[5] Benyamini Y, Roziner I, Goldbourt U, Drory Y, Gerber Y, Israel Study Group on First Acute Myocardial Infarction (2013). Depression and anxiety following myocardial infarction and their inverse associations with future health behaviors and quality of life. Ann Behav Med.

[6] Davidson KW, Rieckmann N, Clemow L, Schwartz JE, Shimbo D, Medina V, Albanese G, Kronish I, Hegel M, Burg MM (2010). Enhanced depression care for patients with acute coronary syndrome and persistent depressive symptoms: coronary psychosocial evaluation studies randomized controlled trial. Arch Intern Med.

[7] Huffman JC, Mastromauro CA, Beach SR, Celano CM, DuBois CM, Healy BC, Suarez L, Rollman BL, Januzzi JL (2014). Collaborative care for depression and anxiety disorders in patients with recent cardiac events: the Management of Sadness and Anxiety in Cardiology (MOSAIC) randomized clinical trial. JAMA Intern Med.

[8] Richards SH, Anderson L, Jenkinson CE, Whalley B, Rees K, Davies P, Bennett P, Liu Z, West R, Thompson DR, Taylor RS (2017). Psychological interventions for coronary heart disease. Cochrane Database Syst Rev.

[9] Dickens C, Cherrington A, Adeyemi I, Roughley K, Bower P, Garrett C, Bundy C, Coventry P (2013). Characteristics of psychological interventions that improve depression in people with coronary heart disease: a systematic review and meta-regression. Psychosom Med.

[10] Cowie MR, Chronaki CE, Vardas P (2013). e-Health innovation: time for engagement with the cardiology community. Eur Heart J.

[11] Rebello TJ, Marques A, Gureje O, Pike KM (2014). Innovative strategies for closing the mental health treatment gap globally. Curr Opin Psychiatry.

[12] Andrews G, Cuijpers P, Craske MG, McEvoy P, Titov N (2010). Computer therapy for the anxiety and depressive disorders is effective, acceptable and practical health care: a meta-analysis. PLoS One.

[13] van Beugen S, Ferwerda M, Hoeve D, Rovers MM, Spillekom-van Koulil S, van Middendorp H, Evers AW (2014). Internet-based cognitive behavioral therapy for patients with chronic somatic conditions: a meta-analytic review. J Med Internet Res.

[14] Glozier N, Christensen H, Naismith S, Cockayne N, Donkin L, Neal B, Mackinnon A, Hickie I (2013). Internet-delivered cognitive behavioural therapy for adults with mild to moderate depression and high cardiovascular disease risks: a randomised attention-controlled trial. PLoS One.

[15] Norlund F, Olsson EM, Burell G, Wallin E, Held C (2015). Treatment of depression and anxiety with internet-based cognitive behavior therapy in patients with a recent myocardial infarction (U-CARE Heart): study protocol for a randomized controlled trial. Trials.

[16] Zigmond AS, Snaith RP (1983). The Hospital Anxiety and Depression Scale. Acta Psychiatr Scand.

[17] Svanborg P, Asberg M (2001). A comparison between the Beck Depression Inventory (BDI) and the self-rating version of the Montgomery Asberg Depression Rating Scale (MADRS). J Affect Disord.

[18] Andersson G, Estling F, Jakobsson E, Cuijpers P, Carlbring P (2011). Can the patient decide which modules to endorse? An open trial of tailored internet treatment of anxiety disorders. Cogn Behav Ther.

[19] Paxling B, Lundgren S, Norman A, Almlöv J, Carlbring P, Cuijpers P, Andersson G (2013). Therapist behaviours in internet-delivered cognitive behaviour therapy: analyses of e-mail correspondence in the treatment of generalized anxiety disorder. Behav Cogn Psychother.

[20] Jernberg T, Attebring MF, Hambraeus K, Ivert T, James S, Jeppsson A, Lagerqvist B, Lindahl B, Stenestrand U, Wallentin L (2010). The Swedish Web-system for enhancement and development of evidence-based care in heart disease evaluated according to recommended therapies (SWEDEHEART). Heart.

[21] Bjelland I, Dahl AA, Haug TT, Neckelmann D (2002). The validity of the Hospital Anxiety and Depression Scale. An updated literature review. J Psychosom Res.

[22] van Ballegooijen W, Riper H, Cuijpers P, van Oppen P, Smit JH (2016). Validation of online psychometric instruments for common mental health disorders: a systematic review. BMC Psychiatry.

[23] Holländare F, Andersson G, Engström I (2010). A comparison of psychometric properties between internet and paper versions of two depression instruments (BDI-II and MADRS-S) administered to clinic patients. J Med Internet Res.

[24] Manos RC, Kanter JW, Luo W (2011). The behavioral activation for depression scale-short form: development and validation. Behav Ther.

[25] Eifert GH, Thompson RN, Zvolensky MJ, Edwards K, Frazer NL, Haddad JW, Davig J (2000). The Cardiac Anxiety Questionnaire: development and preliminary validity. Behav Res Ther.

[26] Păsărelu CR, Andersson G, Bergman Nordgren L, Dobrean A (2017). Internet-delivered transdiagnostic and tailored cognitive behavioral therapy for anxiety and depression: a systematic review and meta-analysis of randomized controlled trials. Cogn Behav Ther.

[27] Wallin E, Norlund F, Olsson EM, Burell G, Held C, Carlsson T (2018). Acceptability of internet-based cognitive behavioral therapy for adults with depression and anxiety after a myocardial infarction: a mixed methods study. J Med Internet Res.

[28] van Buuren S, Groothuis-Oudshoorn K (2011). mice: multivariate imputation by chained equations in R. J Stat Softw.

[29] Rubin D (1987). Multiple Imputation for Nonresponse in Surveys.

[30] R Core Team, R Development Core Team (2015). R: A Language and Environment for Statistical Computing.

[31] Messerli-Bürgy N, Barth J, Berger T (2012). The InterHerz project--a web-based psychological treatment for cardiac patients with depression: study protocol of a randomized controlled trial. Trials.

[32] Musiat P, Goldstone P, Tarrier N (2014). Understanding the acceptability of e-mental health--attitudes and expectations towards computerised self-help treatments for mental health problems. BMC Psychiatry.

[33] Wallin EE, Mattsson S, Olsson EM (2016). The preference for internet-based psychological interventions by individuals without past or current use of mental health treatment delivered online: a survey study with mixed-methods analysis. JMIR Ment Health.

[34] Ogmundsdottir Michelsen H, Hagstrom E, Sjolin I, Schlyter M, Kiessling MA, Held C, Hag E, Nilsson L, Schiopu A, Zaman MJ, Leosdottir M (2017). Swedish cardiac rehabilitation programmes; a descriptive nationwide analysis - the perfect CR study. Eur Heart J (Supplement 1).

[35] Newby JM, Mackenzie A, Williams AD, McIntyre K, Watts S, Wong N, Andrews G (2013). Internet cognitive behavioural therapy for mixed anxiety and depression: a randomized controlled trial and evidence of effectiveness in primary care. Psychol Med.

[36] Allen AR, Newby JM, Mackenzie A, Smith J, Boulton M, Loughnan SA, Andrews G (2016). Internet cognitive-behavioural treatment for panic disorder: randomised controlled trial and evidence of effectiveness in primary care. BJPsych Open.

[37] Knowles SE, Lovell K, Bower P, Gilbody S, Littlewood E, Lester H (2015). Patient experience of computerised therapy for depression in primary care. Br Med J Open.

[38] Andersson G, Cuijpers P (2009). Internet-based and other computerized psychological treatments for adult depression: a meta-analysis. Cogn Behav Ther.

[39] O'Mahen HA, Richards DA, Woodford J, Wilkinson E, McGinley J, Taylor RS, Warren FC (2014). Netmums: a phase II randomized controlled trial of a guided Internet behavioural activation treatment for postpartum depression. Psychol Med.

[40] Orth-Gomér K, Schneiderman N, Wang H, Walldin C, Blom M, Jernberg T (2009). Stress reduction prolongs life in women with coronary disease: the Stockholm Women's Intervention Trial for Coronary Heart Disease (SWITCHD). Circ Cardiovasc Qual Outcomes.

[41] Gulliksson M, Burell G, Vessby B, Lundin L, Toss H, Svärdsudd K (2011). Randomized controlled trial of cognitive behavioral therapy vs standard treatment to prevent recurrent cardiovascular events in patients with coronary heart disease: Secondary Prevention in Uppsala Primary Health Care project (SUPRIM). Arch Intern Med.

[42] Blumenthal JA, Sherwood A, Smith PJ, Watkins L, Mabe S, Kraus WE, Ingle K, Miller P, Hinderliter A (2016). Enhancing cardiac rehabilitation with stress management training: a randomized, clinical efficacy trial. Circulation.

[43] Hedman E, Ljótsson B, Kaldo V, Hesser H, El AS, Kraepelien M, Andersson E, Rück C, Svanborg C, Andersson G, Lindefors N (2014). Effectiveness of Internet-based cognitive behaviour therapy for depression in routine psychiatric care. J Affect Disord.

[44] Lindner P, Nyström MB, Hassmén P, Andersson G, Carlbring P (2015). Who seeks ICBT for depression and how do they get there? Effects of recruitment source on patient demographics and clinical characteristics. Internet Interv.

[45] Crabb RM, Cavanagh K, Proudfoot J, Learmonth D, Rafie S, Weingardt KR (2012). Is computerized cognitive-behavioural therapy a treatment option for depression in late-life? A systematic review. Br J Clin Psychol.

[46] Christensen H, Griffiths KM, Farrer L (2009). Adherence in internet interventions for anxiety and depression. J Med Internet Res.

[47] Hanssen TA, Nordrehaug JE, Eide GE, Bjelland I, Rokne B (2009). Anxiety and depression after acute myocardial infarction: an 18-month follow-up study with repeated measures and comparison with a reference population. Eur J Cardiovasc Prev Rehabil.

[48] [No authors listed] (1999). ICH harmonised tripartite guideline. Statistical principles for clinical trials. International Conference on Harmonisation E9 Expert Working Group. Stat Med.

[49] Hoffmann TC, Glasziou PP, Boutron I, Milne R, Perera R, Moher D, Altman DG, Barbour V, Macdonald H, Johnston M, Lamb SE, Dixon-Woods M, McCulloch P, Wyatt JC, Chan AW, Michie S (2014). Better reporting of interventions: template for intervention description and replication (TIDieR) checklist and guide. Br Med J.

[50] Bower P, Kontopantelis E, Sutton A, Kendrick T, Richards DA, Gilbody S, Knowles S, Cuijpers P, Andersson G, Christensen H, Meyer B, Huibers M, Smit F, van Straten A, Warmerdam L, Barkham M, Bilich L, Lovell K, Liu ET (2013). Influence of initial severity of depression on effectiveness of low intensity interventions: meta-analysis of individual patient data. Br Med J.

[51] Ennis L, Wykes T (2013). Impact of patient involvement in mental health research: longitudinal study. Br J Psychiatry.

