# Supplementary figures and images for: Internet-Based Cognitive Behavioral Therapy for Symptoms of Depression and Anxiety Among Patients With a Recent Myocardial Infarction: The U-CARE Heart Randomized Controlled Trial
Source: J Med Internet Res. 2018 Mar 8;20(3):e88. doi: 10.2196/jmir.9710 (PMC5874001; doi:10.2196/jmir.9710)

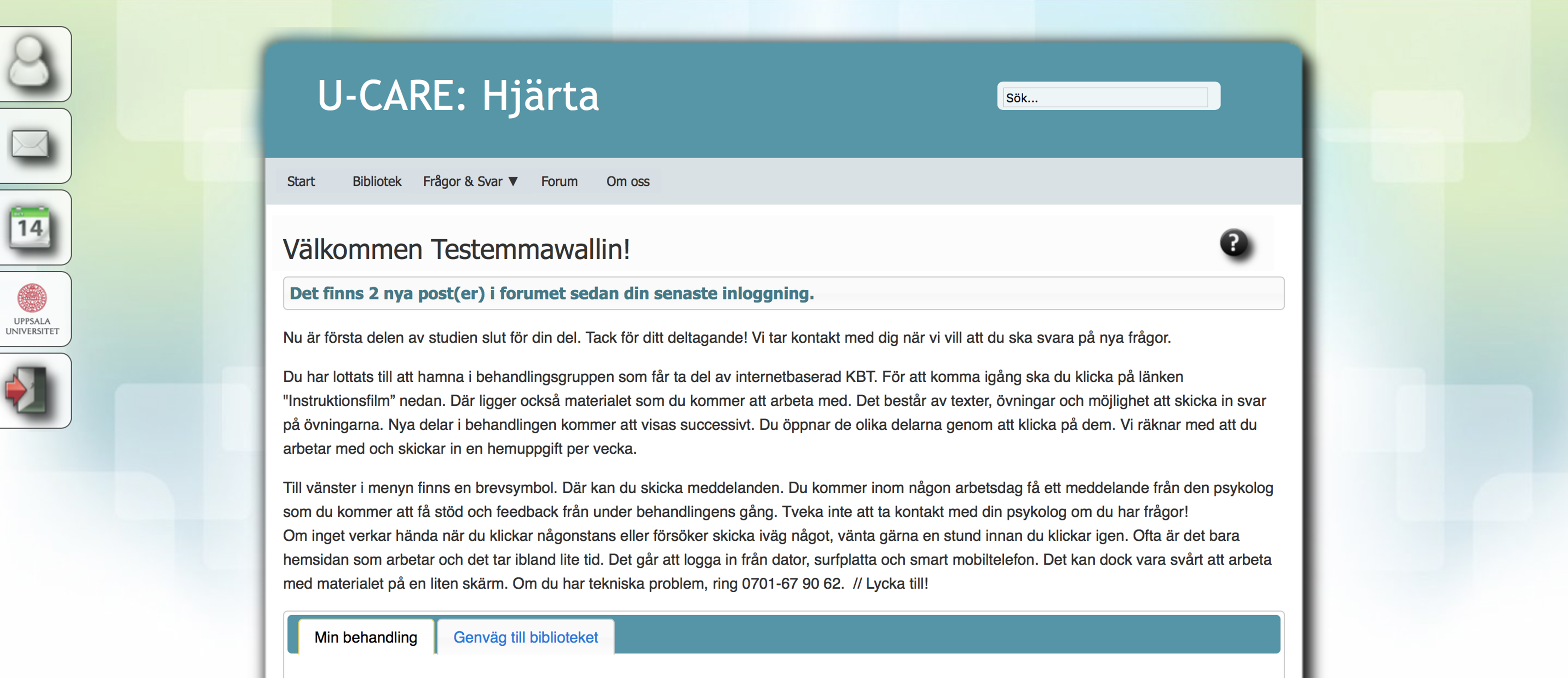

Supplement: Multimedia Appendix 2 [file jmir_v20i3e88_app2.jpg]
